# Development of an HPLC-FLD Method for Estradiol and Metabolites: Application of Solid-Phase Microextraction

**DOI:** 10.3390/ijms26136194

**Published:** 2025-06-27

**Authors:** Anna Kaliszewska, Piotr Struczyński, Tomasz Bączek, Lucyna Konieczna

**Affiliations:** 1 Department of Pharmaceutical Chemistry, Medical University of Gdańsk, Hallera 107, 80-416 Gdańsk, Poland; anna.kaliszewska@gumed.edu.pl (A.K.); piotr.struczynski@gumed.edu.pl (P.S.); or tomasz.baczek@upsl.edu.pl (T.B.); 2 Department of Nursing and Medical Rescue, Institute of Health Sciences, Pomeranian University in Słupsk, 76-200 Słupsk, Poland

**Keywords:** estrogen, derivatization procedure, fluorescence detection

## Abstract

Estrogens are potent hormones involved in numerous physiological and pathological processes. Their typically low concentrations in biological samples necessitate highly sensitive analytical methods for accurate quantification. This study presents a high-performance liquid chromatography with fluorescence detection (HPLC-FLD) method for quantifying estradiol and its metabolites in blood serum and saliva. Analytes were extracted using solid-phase microextraction with a divinylbenzene sorbent and methanol as the desorption agent. FLD was performed after the derivatization of the analytes with dansyl chloride. Separation was achieved on a Poroshell 120 EC-C18 column (2.1 × 100 mm, 2.7 µm) at 50 °C using water with 0.1% formic acid and methanol as the mobile phase at 0.5 mL/min. A gradient elution increased the methanol concentration from 76% to 100% over 0–8 min, then it returned to 76% at 8.1 min and was held until 11 min had passed. Detection was at λ_EX_ 350 nm and λ_EM_ 530 nm. Good linearity was observed for estradiol, 2-hydroxyestradiol, and 2-methoxyestradiol (10–300 ng/mL; R^2^ = 0.9893–0.9995). The LOQ for all analytes was 10 ng/mL. Solid-phase microextraction (SPME) offered advantages over liquid–liquid extraction. The method is suitable for quantifying estrogens in the 10 ng/mL–1 µg/mL range.

## 1. Introduction

### 1.1. Estrogen in Cancer Development

Prolonged estrogen exposure increases cancer risk, most notably breast cancer: early menarche and late menopause raise the risk, while later menarche and early menopause lower it [1,2]. Although estrogen levels fall after menopause, most breast cancers occur then [3]. Estrogens drive breast epithelial proliferation, heightening DNA replication errors and mitochondrial ROS production, and their metabolism to quinones further promotes mutagenesis, especially when DNA repair is inhibited [3,4,5].

Beyond the breast, estrogens elevate ovarian cancer risk—both endogenous and HRT-derived—whereas contraceptives reduce it by suppressing ovulation; each additional five years of ovulatory cycles (~60 cycles) increases the risk by 14% [6]. Estrogen–progesterone imbalance also underlies endometrial cancer development [7,8]. In men, higher free estradiol and estradiol–testosterone ratios correlate with prostate cancer risk as testosterone declines with age [9], and elevated estradiol levels predict poorer survival in non–small-cell lung cancer [10].

### 1.2. Estrogen Metabolites

Most of the literature on the role of estrogens in cancer development focuses on the most potent compounds in this group, commonly referred to as parent estrogens—estrone and estradiol. These hormones are metabolized via three primary pathways (2-, 4-, and 16-hydroxylation pathways), resulting in metabolites with distinct biological activities compared to E1 and E2 [11]. Notably, metabolites formed through the 2-hydroxylation pathway are thought to have protective effects against cancer development and progression [12]. Fotsis et al. [13] demonstrated that 2-methoxyestradiol (2-MeOE2), a downstream product of this pathway, inhibits tumor angiogenesis, while Chander et al. [14] showed its ability to suppress cancer cell proliferation. Recent studies suggest that 2-MeOE2 may have therapeutic potential across a range of malignancies [15], including prostate cancer [16], osteosarcoma [17], ovarian cancer [18], non-small-cell lung cancer [19], and breast cancer [20].

Another metabolite associated with reduced cancer risk is 2-hydroxyestardiol (2-OHE2), which also exhibits strong anti-proliferative and anti-angiogenic properties [13,21]. In a case–control study, Falk et al. [11] indicated that enhanced metabolism through the 2-hydroxylation pathway may lower the risk of postmenopausal breast cancer. However, data on the therapeutic applications of 2-OHE2 remain limited, and further investigation is warranted to assess its potential in cancer treatment.

### 1.3. Estrogen Derivatization

Estrogens are biologically active compounds that circulate in the human body at extremely low concentrations, typically in the pictogram-per-milliliter range [22]. Accurate quantification of these hormones is essential for the diagnosis and monitoring of endocrine disorders. As such, the development of highly sensitive and precise analytical methods for estrogen determination is of considerable importance.

FLD and mass spectrometry (MS) are widely employed analytical techniques due to their low limits of detection and quantification, along with high sensitivity [23]. However, most steroid hormones, including estrogens, lack intrinsic fluorescence, which precludes direct analysis by FLD. To address this limitation, derivatization with fluorophore-containing reagents is commonly applied. Similarly, in LC-MS, derivatization is often recommended, as estrogens lack functional groups that readily ionize under standard electrospray ionization conditions [24].

### 1.4. Quantitative Analysis of Estrogen in Biological Samples

Numerous studies have explored different methodologies for the quantification of estrogens in biological samples. These studies vary in terms of their sample matrix, preparation techniques, extraction solvents, derivatization agents, and detection methods. A review of the literature indicates that blood serum is the most frequently analyzed matrix [25], although other biofluids such as saliva [26], plasma [27], urine [28], and cerebrospinal fluid [29] have also been investigated.

Liquid–liquid extraction (LLE) remains the most commonly employed sample preparation technique. Among the extraction solvents, methyl tert-butyl ether (MTBE) is the most widely used [26], though dichloromethane [30], diethyl ether [31], toluene [32], and ethyl acetate [29] have also been utilized. Dansyl chloride (DNS-Cl) is the predominant derivatization agent [33], although alternatives such as *p*-nitrobenzoyl chloride [34] have also been applied. Notably, some studies have omitted the derivatization step entirely [28].

LC–MS/MS is widely preferred for its superior sensitivity, routinely achieving detection and quantification limits in the picogram-per-milliliter range. For example, Mao et al. [34] reported a LOD of 2.7 ng/mL for estradiol in urine using HPLC-FLD after SPE and p-nitrobenzoyl derivatization, whereas Kaski-Rahkonen et al. [25] achieved an LLOQ of 0.5 pg/mL for estradiol in serum by combining MTBE liquid–liquid extraction, derivatization with 1,2-dimethylimidazole-5-sulfonyl chloride, and LC–MS/MS.

Among E2, 2-MeOE2, and 2-OHE2, the lowest LOQ values are consistently reported for estradiol, while 2-OHE2 generally exhibits the highest. In the study by Li et al. [35], the LLOQs for E2, 2-OHE2, and 2-MeOE2 were 2.5 pg/mL, 50 pg/mL, and 4.4. pg/mL, respectively. A summary of key quantitative estrogen analyses is provided in Table 1.

### 1.5. HPLC-FLD

LC-MS has been widely employed for the analysis of estrogens in biological samples, as evidenced by numerous studies in the available literature [25,26,27,30,32,33,35]. In contrast, only a limited number of publications describe the use of LC-FLD for this purpose, with most studies dating back to the 1970s [36,37]. Moreover, these earlier studies primarily focus on the fluorescence-based analysis of pharmaceutical formulations rather than biological matrices. More recent research has explored the application of FLD in environmental sample analysis [38].

Although LC-MS offers excellent sensitivity and a low LOD, this technique has certain limitations. Mass spectrometers are not widely accessible, and their high acquisition and maintenance costs, along with potential instrument downtime due to technical failures, must be considered.

This study aimed to optimize an HPLC-FLD method coupled with solid-phase microextraction (SPME) for the quantification of estradiol and its metabolites in biological samples. The proposed method is evaluated as a potential alternative to LC-MS and LC-MS/MS, offering a more accessible and cost effective approach to estrogen analysis.

## 2. Results

### 2.1. Optimization of the Chromatographic Conditions

Initial analyses were performed with the chromatographic column thermostated at 70 °C, following the conditions described by Schmidt et al. [37]. However, as this temperature is relatively high, lower column temperatures (50 °C and 60 °C) were subsequently evaluated. Reducing the column temperature led to shorter retention times without significantly compromising the signal intensity. As a result, 50 °C was selected as the optimal temperature for all subsequent analyses.

The separation of the three estrogenic compounds analyzed in this study was challenging due to their structural similarities. Given the lipophilic nature of estradiol and its metabolites, reverse-phase liquid chromatography was employed. Several combinations of organic solvents, stationary phases, and mobile phase gradients were assessed to optimize chromatographic resolution. The tested conditions are summarized in Table 2.

Optimal separation was achieved using an InfinityLab Poroshell 120 EC-C18 column with methanol as the organic component of the mobile phase, under the gradient conditions outlined in Table 2.

### 2.2. Optimization of Derivatization Procedure

The efficiency of dansylation is strongly influenced by several reaction parameters, particularly the reaction temperature and duration. To optimize these conditions, 900 µL of a working standard solution of E2 at a concentration of 1 µg/mL was evaporated to dryness. The dry residue was reconstituted with 100 µL of NaHCO_3_ buffer and 200 µL of DNS-Cl solution.

In the first experiment, samples was heated at 40 °C, 60 °C, 80 °C, and 100 °C for 15 min to determine the optimal reaction temperature. In a subsequent experiment, the reaction temperature was fixed at 80 °C, and the samples were heated for, 5, 10, 15, and 20 min to evaluate the effect of reaction time.

The highest signal intensity was observed for the derivatives formed by heating the reaction mixture at 80 °C for 15 min, indicating this as the optimal condition for dansylation under the tested parameters.

### 2.3. Stability of Dansylated Estrogens

**Figure 1:**
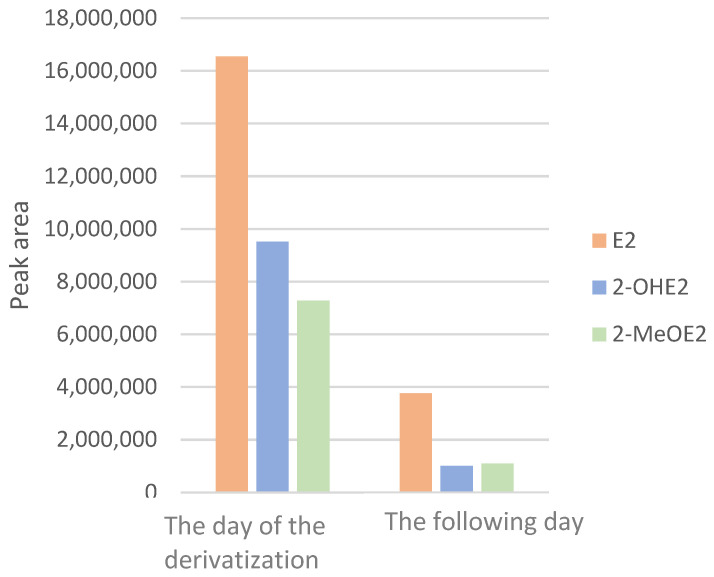
Stability of analytical signals depending on when the samples were derivatized: estradiol (orange), 2-hydroxyestradiol (blue), 2-methoxyestradiol (green).

The stability of the dansyl derivatives of E2 and its metabolites was evaluated by analyzing identical samples of all three compounds over consecutive days. A marked decrease in signal intensity was observed in samples analyzed one day after derivatization, compared to those analyzed on the day of derivatization. Consequently, to ensure optimal signal intensity and analytical reliability, all samples were derivatized immediately prior to analysis (Figure 1).

### 2.4. Liquid–Liquid Extraction Optimization

Serum samples were prepared by spiking 180 µL Bovine blood serum with 20 µL of a standard working solution containing E2, 2-OHE2, and 2-MeOHE2 at a concentration of 10 µg/mL (resulting in a final concentration of 1 µg/mL in the samples). The samples were then diluted 2-fold and 4-fold with deionized water.

LLE was performed using methanol, methanol (MeOH):formic acid (FA) (100:0.1, *v*/*v*), and DCHM as extractants. The results of these extractions are presented in Table 3. Based on the analysis, 4-fold dilution and extraction with DCHM were determined to be the most suitable conditions.

### 2.5. Solid-Phase Microextraction Optimization

Optimization of the SPME procedure began with the selection of the sorbent material and desorption solvent. Sample preparation involved spiking 180 µL of Bovine serum with 20 µL of a working estradiol at a concentration of 10 µg/mL, resulting in a final analyte concentration of 1 µg/mL.

**Figure 2:**
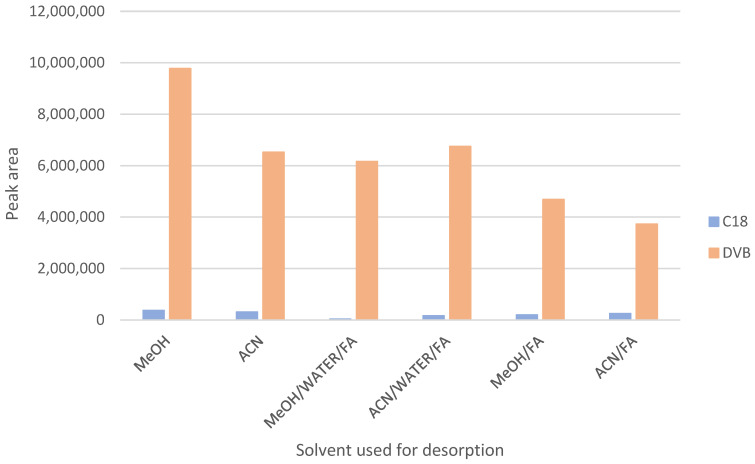
Selection of sorbents and desorption agents for solid-phase microextraction; MeOH—methanol; ACN—acetonitrile; FA—formic acid.

To determine the optimal extraction conditions, SPME was performed using two types of sorbents—C18 and divinylbenzene (DVB)—in combination with six different desorption solvents: MeOH, acetonitrile (ACN), MeOH:H_2_O:FA (50:50:0.1, *v*/*v*/*v*), ACN:H_2_O:FA (50:50:0.1, *v*/*v*/*v*), MeOH:FA (100:0.1, *v*/*v*), and ACN:FA (100:0.1, *v*/*v*). Among the tested combinations, the highest signal intensities were obtained using DVB-coated blades and methanol as the desorption solvent (Figure 2).

**Figure 3:**
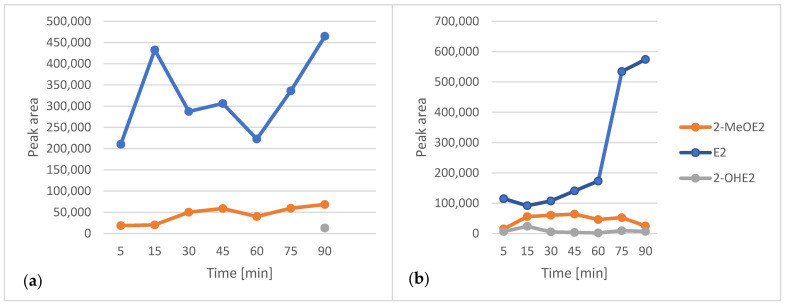
Examination of SPME sorption (**a**) and desorption (**b**) times.

Following sorbent and solvent optimization, the sorption and desorption times were systematically evaluated. Seven samples underwent SPME simultaneously, with sorption times of 5, 15, 30, 45, 60, 75, and 90 min, while the desorption time was held constant at 90 min. In a parallel experiment, the effect of varying desorption time was investigated independently. The optimal extraction efficiency was achieved with a sorption time of 90 min and a desorption time of 90 min, as these conditions yielded the highest signal intensities (Figure 3).

### 2.6. Calibration Curves

The first step in calibrating the method involved assessing the linearity between analyte concentration and signal response across a higher concentration range. Standard working solutions of estrogenic metabolites in ethanol were prepared at five concentration levels (10–1000 ng/mL). The analysis confirmed good linearity across this entire range.

Calibration curves for biological matrices were constructed by spiking bovine serum or saliva (obtained from a healthy volunteer) with the standard working solutions of the analytes at eleven concentration levels (0.05–300 ng/mL). The samples were processed using both LLE and SMPE, followed by derivatization and analysis.

Good linearity was observed in the concentration range of 10–300 ng/mL, with correlation coefficients (R^2^) ranging from 0.9893 to 0.9995. The LOQ for all three analytes was established at 10 ng/mL. The calibration curve equations, R^2^ values, and accuracy data for serum and saliva samples are summarized in Table 4.

### 2.7. LLE and SPME Comparison

**Figure 4:**
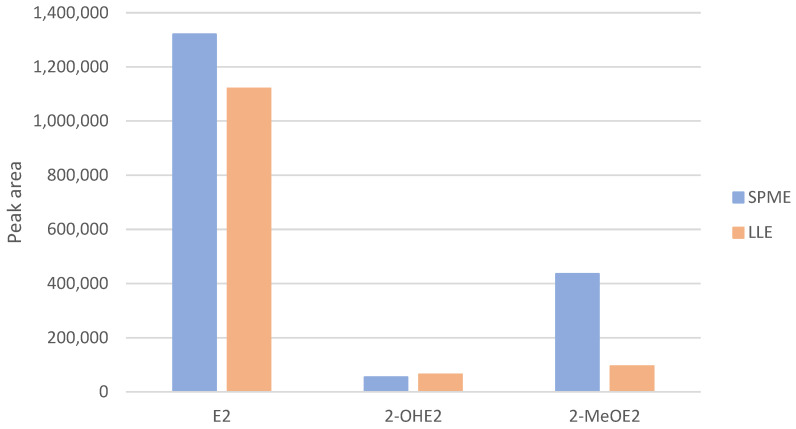
Comparison of the results of serum sample analyses following SPME and LLE.

Figure 4 presents a comparison of the analytical results obtained from serum samples extracted using SPME and LLE. The graph illustrates the differences in signal intensities for the analyzed compounds, highlighting the superior extraction efficiency of the SPME method. These results confirm the suitability of SPME as a reliable and effective alternative to traditional LLE for the quantification of estrogenic metabolites in biological matrices.

### 2.8. LC-MS Analysis

To assess the performance of LC-MS combined with SPME for estrogen detection, saliva samples prepared for calibration (following LLE) were analyzed using LC-MS. The results are summarized in Table 5. Excellent linearity was observed across the concentration range of 0.05–300 ng/mL for E2, and 1–300 ng/mL for 2-OHE2 and 2-MeOE2.

### 2.9. Patient Samples

Reliable quantification of estrogens in biological samples using HPLC-FLD proved challenging, as the achieved LOQ exceeded the expected estrogen concentrations in the analyzed matrices. In some cases, the analytical signals for E2 and its metabolites were too weak to enable precise and accurate quantification. Conversely, other samples displayed disproportionately large peak areas, suggesting estrogen levels that were significantly higher than anticipated. These inconsistencies may be attributed to inadequate sample purification prior to analysis, potentially leading to matrix effects or the presence of interfering substances.

## 3. Discussion

The development of an HPLC-FLD method coupled with SPME for the quantification of estradiol and its metabolites represents a deliberate effort to address the accessibility and cost barriers associated with LC-MS/MS. While the method demonstrated robustness in specific applications, its performance must be contextualized within recent advancements in estrogen analysis.

The linearity (R^2^ = 0.9893–0.9995) and precision (accuracy: 96.05–101.85%) observed for ethanol-based standards within the 10–300 ng/mL range underscore the reliability of the chromatographic and derivatization protocols. However, the LOQ of 10 ng/mL, while adequate for pharmaceutical quality control, remains insufficient for clinical diagnostics, where endogenous estrogen levels often fall below 1 ng/mL. This limitation is inherent to FLD, which cannot match the picogram-level sensitivity of modern LC-MS/MS platforms. For instance, Cerrato et al. [39] recently reported an online monolithic extraction method coupled with LC-MS/MS, achieving an LOQ of 0.008 ng/mL for E2 in serum through a fully automated workflow. Similarly, Yi et al. [40] demonstrated an aptamer-based enrichment strategy with LC-MS/MS, attaining LOQs of 0.02 ng/mL for E2 via selective aptamer-functionalized magnetic beads. These studies highlight the growing emphasis on automation, sensitivity, and matrix resistance in estrogen analysis—areas where fluorescence-based methods face inherent constraints.

The SPME approach described here offers distinct advantages over traditional LLE, including reduced solvent consumption (~70% less) and improved chromatographic clarity. However, emerging techniques such as monolithic columns [39] and aptamer-mediated extraction [40] set new benchmarks for efficiency and specificity. The monolithic poly(propargyl amine) polymer developed by Cerrato et al. [39] enabled near-complete automation, with recoveries > 94% and minimal manual intervention. In contrast, Yi et al. [40] leveraged a class-specific aptamer to selectively enrich estrogens, mitigating matrix effects and achieving recoveries of 94.9–107.7%. These innovations underscore the potential of combining advanced materials or biorecognition elements with MS to address challenges in complex biofluids—a domain where FLD struggles due to interference and derivative instability.

Despite its limitations in biological matrices, the method’s broad dynamic range (10–1000 ng/mL) and reproducibility position it as a pragmatic tool for pharmaceutical applications. For example, it could be utilized in the batch testing of topical estrogen formulations or stability studies, where analyte concentrations are intentionally elevated. This aligns with historical uses of HPLC-FLD in drug quality control while modernizing workflows through SPME’s eco-friendly profile. The reduced solvent consumption and potential for parallel sample processing further enhance its suitability for high-throughput environments, resonating with green chemistry initiatives emphasized in both cited studies.

To bridge the sensitivity gap, future iterations could integrate SPME with post-column photochemical reactors or advanced derivatization agents (e.g., 1,2-dimethylimidazole sulfonyl chloride) to enhance fluorescence quantum yields. Hybrid workflows inspired by Cerrato et al. [39]—such as coupling SPME with online LC-MS platforms—might improve throughput and reproducibility. Additionally, exploring aptamer-functionalized SPME fibers, as demonstrated by Yi et al. [40], could enhance selectivity for estrogen metabolites in complex matrices. Extending the method to Phase II metabolites (e.g., sulfated estrogens) would broaden its relevance in metabolic studies, while automated SPME systems could streamline workflows for resource-limited laboratories.

The environmental impact of the proposed method, characterized by miniaturized synthesis and reduced solvent use, aligns with trends in sustainable analytical chemistry. However, the dominance of LC-MS/MS in clinical diagnostics remains unchallenged due to its unparalleled sensitivity and multiplexing capabilities. The work by Cerrato et al. [39] and Yi et al. [40] exemplifies how automation and selective enrichment strategies are redefining analytical workflows, emphasizing the need for context-specific method development. While the HPLC-FLD/SPME approach does not replace LC-MS/MS, it offers a complementary solution for scenarios prioritizing cost, speed, and sustainability over ultra-trace detection.

## 4. Materials and Methods

### 4.1. Chemicals

E2, 2-OHE2, DNS-Cl, formic acid (FA), and Adult Bovine Serum were purchased from Sigma-Aldrich (St. Louis, MO, USA), while 2-OHE2 was obtained from Cayman Chemical (Ann Arbor, MI, USA). Ethanol (EtOH), and methanol (MeOH), acetone, acetonitrile (ACN), all HPLC grade, were sourced from Merck (Darmstadt, Germany). Dichloromethane (DCHM) was purchased from Honeywell (Morristown, NJ, USA). Sodium bicarbonate and sodium hydroxide, both of analytical reagent grade, were obtained from POCH (Gliwice, Poland). Deionized water was purified using a Milli-Q Direct-Q^®^ 3 UV system (Millipore, Bedford, MA, USA).

Stock standard solutions of estrogens were prepared in ethanol at a concentration of 1 mg/mL and stored at −4 °C. The DNS-Cl solution was freshly prepared every two days by dissolving 10 mg of the reagent in 10 mL of acetone and stored at −4 °C to preserve reactivity. A sodium bicarbonate buffer was prepared by dissolving 8.69 g of NaHCO_3_ in an appropriate volume of deionized water, followed by the addition of 16 mL of 5 M NaOH to adjust the pH to 9.5. The final volume was brought to 100 mL with deionized water.

### 4.2. Instrumentation and Chromatographic Conditions

Chromatographic analyses were performed using a Shimadzu Prominence LC-2030C 3D System (Shimadzu, Kyoto, Japan), equipped with a pump, autosampler, column oven, and a PDA detector from the LC-2030 series, complemented by a fluorescence detector (RF-20AXS). Separation was carried out on an InfinityLab Poroshell 120 EC-C18 column (100 × 2.1 mm, 2.7 µm; Agilent Technologies, Palo Alto, CA, USA). Data acquisition and processing were performed using Shimadzu LabSolutions software (version 5.3).

The column temperature was maintained at 50 °C. The mobile phase consisted of water with 0.1% formic acid (Phase A) and methanol (Phase B). Gradient elution was applied, with the methanol content increasing from 76% to 100% over 0–8 min, followed by a rapid return to 76% between 8 and 8.1 min, which was maintained until the end of the run at 11 min. The flow rate was set at 0.5 mL/min. Analytes were detected by fluorescence with excitation and emission wavelengths of λEX 282 nm and λEM 530 nm, respectively. SPME was performed using a 96-well plate system (AEK-SH10 SA, Caterpillar, Deerfield, IL, USA).

LC-MS analysis was conducted using an Agilent 1260 Infinity HPLC system, including a pump and autosampler from the 1260 series, coupled with a single quadrupole mass spectrometer (Agilent Technologies, Palo Alto, CA, USA). The same InfinityLab Poroshell 120 EC-C18 column was used. Data processing was carried out with Agilent ChemStation software (version B.04.03). The column temperature, mobile phase composition, and flow rate were identical to those used in the HPLC-FLD analysis. The gradient elution was slightly modified, increasing methanol from 80% to 100% between 0 and 8 min, then returning to 80% between 8 and 8.1 min, where it remained until the 11th minute. LC-MS parameters included positive polarity mode, an ion spray voltage of 3000 V, a drying gas flow rate of 10 L/min, a heating block temperature of 350 °C, and a curtain gas pressure of 50 psi.

### 4.3. Samples

Blood serum and saliva samples were obtained from healthy volunteers within the research group, who provided informed consent to participate in this study. Saliva samples were collected using a Salivette kit by instructing participants to remove the swab, place it in their mouth for approximately 15 min, and then return it to the Salivette tube. The samples were subsequently centrifuged at 10,000 rpm for 15 min to separate the saliva, which was then stored at −80 °C until analysis.

### 4.4. Sample Preparation

#### 4.4.1. Liquid–Liquid Extraction

A volume of 200 µL of blood serum was mixed with 600 µL of deionized water in a 5 mL Eppendorf tube, followed by the addition of 3.2 mL of DCHM. For saliva samples, 500 µL of saliva was combined with 4 mL of DCHM in an Eppendorf tube. Both serum and saliva mixtures were vortexed for 15 s, then subjected to rotary mixing at 40 rpm for 20 min. Following this, the samples were centrifuged at 10,000 rpm for 7 min. The upper organic phase was carefully transferred to a new Eppendorf tube and evaporated in a vacuum concentrator at 45 °C. The resulting dry residue was derivatized and subsequently analyzed using HPLC-FLD.

#### 4.4.2. Solid-Phase Microextraction

A volume of 500 µL of blood serum or saliva was transferred into a 1.5 mL Eppendorf tube. For saliva samples, 500 µL of deionized water was added, while for blood serum samples, 500 µL of phosphate-buffer saline (PBS) was used. The mixtures were vortexed for 15 s, followed by rotary mixing at 40 rpm for 20 min,, and then centrifuged at 10,000 rpm for 7 min. A 1 mL aliquot of the supernatant from each sample was transferred to a respective well of a 96-well plate and subjected to SPME.

DVB-covered blades were used as the sorbent and were preconditioned by agitation in a methanol–deionized water (50:50, *v*/*v*) solution for two consecutive 30 min cycles. During the sorption step, the DVB-covered blades were inserted into the sample-filled 96-well plate and agitated at 1000 rpm for 90 min. Following sorption, the blades were briefly rinsed in deionized water before being transferred into a methanol-filled plate for desorption, which was carried out under constant agitation at 1000 rpm for 90 min.

The resulting methanolic extracts were transferred into 1.5 mL Eppendorf tubes and evaporated to dryness at 45 °C using a vacuum concentrator. The dry residue was derivatized and analyzed.

### 4.5. Derivatization Procedure

The dry residue obtained from LLE or SPME was reconstituted in 100 µL of sodium bicarbonate (NaHCO_3_) buffer. Subsequently, 200 µL of DNS-Cl solution was added, and the mixtures were vortexed for 15 s. The samples were then incubated in a heating block at 80 °C for 15 min to facilitate derivatization. After cooling, the samples were carefully centrifuged at 10,000 rpm for 7 min, and the resulting supernatant was carefully transferred into 150 µL glass inserts, which were placed inside glass vials. A 5 µL aliquot of the prepared solution was injected into the HPLC-FLD system for analysis.

## 5. Conclusions

The developed HPLC-FLD method did not allow for the quantification of estrogens in biological samples due to its relatively high LOQ compared to LC-MS/MS. This limitation may be attributed to the lower sensitivity of FLD for dansylated derivatives compared to mass spectrometry. Additionally, the difficulty in accurately determining estrogen concentrations could be related to insufficient sample purification. Further research should focus on optimizing the extraction process to enhance method sensitivity and precision.

Although SPME has been applied previously to estrogen analysis, our work distinguishes itself by combining a readily available divinylbenzene–coated fiber with on-fiber dansyl derivatization and rapid HPLC–FLD for the simultaneous quantification of 17β-estradiol and key metabolites in both serum and saliva. This protocol is intended for pharmaceutical formulation analysis and quality control—for example, the batch verification of estrogen-containing topical or oral products—and has not been validated for direct clinical diagnostics or patient monitoring.

Given the high coefficients of determination observed for ethanol-based estrogen solutions within the concentration range of 10 ng/mL to 1 µg/mL, this method may be applicable for the analysis of samples with higher analyte concentrations, such as pharmaceutical formulations. Furthermore, the HPLC-FLD method demonstrated a broader linear range compared to the approach described by Fishmann [36], who utilized a spectrofluometer for estrogen determination in dosage forms.

LLE remains the most widely used technique for estrogen analysis due to its simplicity. However, SPME presents a viable alternative for sample purification. While SPME requires more specialized instrumentation than LLE, it significantly reduces solvent consumption, making it a more cost-effective and environmentally friendly option in the long term. Additionally, SPME enables the simultaneous processing of multiple samples, increasing analytical efficiency. In this study, the application of SPME resulted in a reduction in the total number of interfering signals and yielded higher R^2^ values compared to LLE, demonstrating its potential advantages in estrogen analysis.

## Figures and Tables

**Table 1:** Examples of studies concerning quantification of estrogen in biological samples.

Biological Material	Extraction	Solvent Used for Extraction	Derivatizing Agent	Quantitative Technique	LLOQ			Source
					E2	2-OHE2	2-MeOE2	
Blood serum	LLE	DCHM	N-methyl-nicotinic acid Nhydroxysuccinimide ester	LC-MS/MS	0.44 ng/mL *	0.69 ng/mL *	0.37 ng/mL *	[30]
Blood serum	LLE	MTBE	1,2-dimethylimidazole-5-sulfonyl chloride	LC-MS/MS	0.5 pg/mL	-	-	[25]
Saliva	SPE	MeOH	-	LC-MS/MS	1 pg/mL	-	-	[26]
Blood plasma	SPE	MeOH:CHI3	3-bromomethyl-propyphenazone	LC-MS/MS	0.3 pg/mL	2.4 pg/mL	0.9 pg/mL	[27]
Blood serum	LLE	MTBE	DNS-Cl	LC-MS/MS	5 pg/mL	-	-	[33]
Blood serum and plasma	LLE	Toluene	-	LC-MS/MS	6.6 pmol/L	-	-	[32]
Blood serum	LLE	MTBE	DNS-Cl	LC-MS/MS (Orbitrap)	2.5 pg/mL	50 pg/mL	4.4 pg/mL	[35]
Urine	SPE	DCHM	*p*-Nitrobenzoyl chloride	HPLC-FLD	2.7 ng/mL *	-	-	[34]
Cerebrospinal fluid	LLE	Ethyl acetate	DNS-Cl	LC-MS/MS	26 pg/mL *	-	-	[29]

* LOD; SPE—solid-phase extraction.

**Table 2:** Optimization of chromatographic conditions for separation of dansylated estradiol and its metabolites.

Column	Organic Phase	Mobile Phase [% Organic Component]		Was the Separation Successful?
Discovery C18 (150 × 4.6 mm, 5 µm)	ACN	50%		No
Discovery C18 (150 × 4.6 mm, 5 µm)	ACN	40%		No
Discovery C18 (150 × 4.6 mm, 5 µm)	ACN	0 min 10 min 10.1 min 14 min	30% 100% 30% 30%	No
Phenomenex C18 (250 × 2.0 mm, 4 µm)	ACN	0 min 12 min 12.1 min 15 min	40% 10% 40% 40%	No
Phenomenex C18 (250 × 2.0 mm, 4 µm)	ACN	0 min 10 min 10.1 min 14 min	50% 100% 50% 50%	No
Phenomenex C18 (250 × 2.0 mm, 4 µm)	ACN	0 min 8 min 8.1 min 11 min	70% 100% 70% 70%	No
InfinityLab Proshell 120 EC-C18 (100 × 2.1 mm, 2.7 µm)	ACN	0 min 8 min 8.1 min 11 min	70% 100% 70% 70%	No
InfinityLab Proshell 120 EC-C18 (100 × 2.1 mm, 2.7 µm)	ACN	0 min 10 min 10.1 min 14 min	70% 100% 70% 70%	No
InfinityLab Proshell 120 EC-C18 (100 × 2.1 mm, 2.7 µm)	ACN	0 min 10 min 10.1 min 14 min	70% 100% 70% 70%	No
InfinityLab Proshell 120 EC-C18 (100 × 2.1 mm, 2.7 µm)	MeOH	0 min 10 min 10.1 min 14 min	70% 100% 70% 70%	No
InfinityLab Proshell 120 EC-C18 (100 × 2.1 mm, 2.7 µm)	**MeOH**	**0 min** **8 min** **8.1 min** **11 min**	**76%** **100%** **76%** **76%**	**Yes**

**Table 3:** Optimization of LLE conditions for blood serum samples.

Scheme	Solvent	Peak Area		
		E2	2-OHE2	2-MeOE2
2-fold	MeOH	805775	57456	6268
	MeOH + 0.1% FA	1382545	10794	15868
	DCHM	1125206	33379	65759
4-fold	MeOH	257809	35261	99468
	MeOH + 0.1% FA	590494	30525	15609
	**DCHM**	**1120908**	**65138**	**95586**

**Table 4:** Calibration curves, coefficients, and accuracy of determination for serum and saliva samples after LLE and SPME.

Analyte	Calibration Curve for Ethanol Solution	Calibration Curve for Serum (SPME)	Calibration Curve for Serum (LLE)	Calibration Curve for Saliva (SPME)	Calibration Curve for Saliva (LLE)
E2	Y = 13,996x + 400,161 R^2^ = 0.9995	Y = 2814.6x – 20,608 R^2^ = 0.9993	Y = 3186.2x – 13,139 R^2^ = 0.9893	Y = 2666.8x + 18,280 R^2^ = 0.9992	Y = 2655.9x + 20,183 R^2^ = 0.994
2-OHE2	Y = 2663x + 31,812 R^2^ = 0.9991	Y = 840.38x + 2684.9 R^2^ = 0.9979	Y = 728.46x − 1882.7 R^2^ = 0.9947	Y = 173.4 + 19,348 R^2^ = 0.9988	Y = 2468.5x + 3884.5 R^2^ = 0.9988
2-MeOE2	Y = 10,759x + 389,649 R^2^ = 0.9985	Y = 311.85x + 4717.3 R^2^ = 0.9995	Y = 314.98x + 836.28 R^2^ = 0.9995	Y = 245.13x + 50,221 R^2^ = 0.9984	Y = 507.78x + 1985.4 R^2^ = 0.9987
	**Accuracy of determinations**				
E2	96.89%	99.12%	101.85%	100.12%	99.32%
2-OHE2	97.49%	99.01%	99.65%	100.93%	99.55%
2-MeOE2	96.05%	99.38%	99.78%	99.21%	100.82%

**Table 5:** Calibration curves and coefficients of determination for saliva samples after LLE analyzed with LC-MS.

	E2	2-OHE2	2-MeOE2
Calibration curve	Y = 4096.47x + 330.64	Y = 890.32x + 3613.9	Y = 2217.1x + 6459.4
R^2^	0.9996	0.993	0.99971

## Data Availability

Data available on request.

## Abbreviations

The following abbreviations are used in this manuscript:

2-MeOE2	2-Methoxyestradiol
2-OHE2	2-Hydroxyestardiol
ACN	Acetonitrile
DNS-Cl	Dansyl chloride
DCHM	Dichloromethane
DVB	Divinylbenzene
E1	Estrone
E2	Estradiol
EtOH	Ethanol
FA	Formic acid
FLD	Fluorescence detector/detection
HPLC	High-performance liquid chromatography
HRT	Hormone replacement therapy
LC-MS	Liquid chromatography-mass spectrometry
LLE	Liquid–liquid extraction
LLOQ	Lower limit of quantification
LOC	Lifetime number of ovulatory cycles
LOD	Limit of detection
LOQ	Limit of quantification
MeOH	Methanol
MTBE	Methyl tert-butyl ether
NSCLC	Non-small-cell lung cancer
PBS	Phosphate-buffered saline
ROS	Reactive oxygen species
SPE	Solid-phase extraction
SPME	Solid-phase microextraction
